# Systematic review and meta-analysis of neonatal outcomes of COVID-19 vaccination in pregnancy

**DOI:** 10.1038/s41390-022-02421-0

**Published:** 2023-01-03

**Authors:** Dingning Zhang, Tingting Huang, Zhihui Chen, Lulu Zhang, Qi Gao, Ge Liu, Jun Zheng, Fangrui Ding

**Affiliations:** 1 Department of Neonatology, Tianjin Central Hospital of Obstetrics and Gynecology, 300000 Tianjin, China; 2 Tianjin Key Laboratory of Human Development and Reproductive Regulation, 300000 Tianjin, China; 3 Department of Neonatology, Nankai University Maternity Hospital, 300000 Tianjin, China

## Abstract

**Background:**

The safety of coronavirus disease 2019 (COVID-19) vaccines during pregnancy is a particular concern. Here, we addressed the neonatal outcomes after maternal vaccination of COVID-19 during pregnancy.

**Methods:**

We systematically searched PubMed, EMBASE, and the WHO COVID-19 Database for studies on neonatal outcomes after maternal COVID-19 vaccination from inception to 3 July 2022. Main neonatal outcomes were related to preterm, small for gestation (SGA), NICU admission, low Apgar score at 5 min (<7), and additional neonatal outcomes such as gestation <34 weeks, low birth weight and some neonatal morbidity were all also analyzed.

**Results:**

A total of 15 studies were included. We found that maternal vaccination during pregnancy was related to the reduction rates of Preterm, SGA, Low Apgar score at 5 min (<7). In addition, there was no evidence of a higher risk of adverse neonatal outcomes after maternal vaccination of COVID-19 during pregnancy, including NICU admission, preterm birth with gestation <34 weeks, low birth weight, very low birth weight, congenital anomalies, and so on.

**Conclusions:**

COVID-19 vaccination in pregnant women does not raise significant adverse effects on neonatal outcomes and is related to a protective effect on some neonatal outcomes.

**Impact:**

- Present study has addressed the neonatal outcomes after maternal vaccination of COVID-19 during pregnancy.
- COVID-19 vaccination in pregnant women does not raise significant adverse effects on neonatal outcomes and is related to a protective effect on some neonatal outcomes.
- The present study could encourage pregnant women to be vaccinated against COVID-19.

## Introduction

Coronavirus disease 2019 (COVID-19) is currently the most concerning health problem worldwide. Since 4 July 2022, there have been 545 million confirmed cases and 6.3 million deaths worldwide, which are reported by the WHO.^1^ COVID-19 during pregnancy has already been reported to be associated with COVID-19-related morbidity and mortality.^2–5^ In addition, including maternal health problems, neonates are also at increased risk of adverse outcomes.^6,7^ Since the first COVID-19 vaccination was initiated by the end of 2020, the effectiveness of vaccination against COVID-19 and prevention of many complications of COVID-19 have been reported by many studies in the general population.^8–11^ Although pregnant women were excluded from the initial COVID-19 vaccination trials due to safety concerns, an increasing number of following data have shown that the COVID-19 vaccination could effectively prevent severe COVID-19 in pregnant women.^12–15^ Regarding the adverse events of COVID-19 during pregnancy as well as the efficacy and safety of COVID-19 vaccination during pregnancy, the WHO recommends the use of several COVID-19 vaccines in pregnant women.^16^ However, COVID-19 vaccination strategies for pregnant women vary from country to country. For example, China does not recommend vaccination against COVID-19, while Israel and Europe encourage pregnant women to be vaccinated.^17–19^ In addition, support from randomized controlled trials (RCTs) on the safety and effectiveness of COVID-19 vaccines as well as the optimal dosing schedule and the timing of vaccination for pregnant women are scarce due to ethical concerns. Thus, vaccination of COVID-19 during pregnancy still needs to be fully understood and explored. In terms of neonates, studies have already proven that antibodies can be transferred across the placenta, while little attention has been given paid to neonatal outcomes.^20,21^ Nevertheless, several individual observational studies exploring the effects of COVID-19 vaccines on pregnancy and perinatal outcomes have included preterm birth, small for gestation (SGA), NICU admission and so on for neonatal outcomes.^22–36^ Due to the importance of neonatal outcomes after maternal pregnancy, there is an immediate need for a systematic review and meta-analysis of published data to clarify differences in neonatal outcomes between COVID-19 vaccination during pregnancy and unvaccination during pregnancy.

## Methods

This systematic review and meta-analysis of neonatal outcomes of COVID-19 vaccination in pregnancy was registered with PROSPERO CRD42022343713. This study was conducted in accordance with the Preferred Reporting Items for Systematic Reviews and Meta-Analyses (PRISMA) statement (Supplemental table 1). Ethical approval and informed consent were not applicable for this work as it was based on previously published data.

### Search strategy

We electronically searched three electronic data sources without language restrictions: PubMed, EMBASE, and the WHO COVID-19 Database, from the inception of the database to 3 July 2022. Various combinations of Medical Subject Headings and free-text aliases were searched by using the following terms: “pregnancy,” “Infant, Newborn,” “COVID-19,” “Vaccines,” and “COVID-19 Vaccines” were searched (details of the search strategy can be found in the Supplementary Methods).

### Study selection

We included studies that reported the neonatal outcomes of COVID-19 vaccination during pregnancy. Studies were screened by learning the title, abstract, or full text. The main outcomes are: preterm, SGA, NICU admission, low Apgar score at 5 min (<7). The following studies were excluded: (1) irrelevant to the subject of the meta-analysis; (2) results reported in studies that did not include any main outcomes; (3) reviews, editorials, conference papers, case reports or animal experiments; and (4) studies that did not include the control group, namely, the unvaccinated pregnant group. Studies were independently screened by two reviewers following the above criteria, and discrepancies were resolved by consensus or with a third reviewer.

### Data extraction

To examine the differences in neonatal outcomes between COVID-19 vaccination and unvaccination during pregnancy. The following data were extracted: country, study design, type of vaccination studied, number of pregnant women vaccinated for COVID-19, number of pregnant women unvaccinated for COVID-19, type of vaccine, number of doses received, timing of administration (trimester), vaccinated and unvaccinated pregnant women with COVID-19 infection, and single birth or not. In addition, the main neonatal outcomes: preterm, SGA, NICU admission, low Apgar score at 5 min (<7); and additional neonatal outcomes: preterm birth with gestation <34 weeks, low birth weight, very low birth weight, congenital anomalies, jaundice, low Apgar score at 1 min (<7), meconium aspiration syndrome, mechanical ventilation, seizures, hypoglycemia, sepsis, encephalopathy, intracranial hemorrhage, transient tachypnea of the newborn, respiratory distress syndrome, umbilical cord blood pH <7.1; were also extracted from studies.

Data extraction was also conducted by two reviewers independently following the context above, and discrepancies were resolved by consensus or with a third reviewer.

### Quality assessment

Each study was scored according to ROBINS-I (observational)^37^ independently by two reviewers, and disagreements were resolved by consensus or with a third reviewer (Supplemental Table 2).

### Statistical analysis

We performed a meta-analysis to pool data from studies. All outcomes were dichotomous outcomes, and we calculated summary odds ratios and mean differences with 95% confidence intervals. Random-effects or fixed-effect models were used to pool the rates and adjusted estimates across studies separately based on the heterogeneity between estimates (*I*²). Fixed-effect models were used if *I*^2^ ≤ 50%, which represents low-to-moderate heterogeneity, and random-effects models were used if *I*^2^ ≥ 50%, representing substantial heterogeneity. An *I*^2^ value of 0–25% represents insignificant heterogeneity, 26–50% low heterogeneity, 51–75% moderate heterogeneity, and >75% high heterogeneity. Statistical analysis was performed by using the Cochrane Collaboration review management software (RevMan 5.4).

## Results

### Search results and basic characteristics

**Fig. 1:**
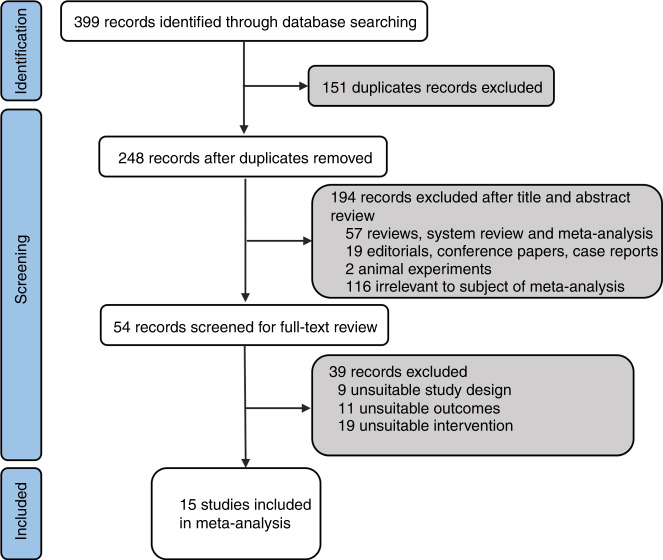
Flow chart of study identification and inclusion.

Based on the search strategy, 146 studies were retrieved from PubMed, 114 studies from Embase, and 139 studies from the WHO COVID-19 Database; a total of 399 studies were identified. A total of 151 duplicates were excluded. According to the titles and abstracts, 194 studies were excluded. After selection criteria were performed based on a full-text review, 39 studies were excluded. Finally, 15 studies were included in this meta-analysis (Fig. 1).^22–36^ Of the 15 studies, 2 case–control studies and 13 cohort studies reported on 90443 vaccinated pregnant women and 265063 unvaccinated pregnant women. The included studies reported data from six countries: Israel, UK, Romania, Canada, US, Sweden, and Norway (Table 1). Most of the pregnant women included in these 15 studies were vaccinated with mRNA vaccines (BNT162b2, mRNA-1273), and a very small portion of the pregnant women were vaccinated with viral vector vaccines and other vaccines (Table 1). Seven of the 15 studies reported details about the number of doses, and 8 of the 15 studies reported details about the trimester at vaccination (Table 1). However, most of these studies did not report neonatal outcomes based on the number of doses and trimester at vaccination. Nine of the 15 studies reported singleton birth, while 7 of these 9 studies excluded multiple births (Table 1). The basic characteristics of the included studies are shown in Table 1.

**Table 1:** Characteristics of the included studies.

Author, year, reference number	Country	Study design	Vaccinated	Unvaccinated	Data collection period	Type of vaccine	Number of doses received	Timing of administration (trimester)	Pregnancy date	Included multiple birth
Beharier et al., 2021^22^	Israel	Case control	92	140	April 2020 to March 2021	BNT162b2	≥1 doses	Second or third trimester	NA	NA
Blakeway et al., 2022^23^	UK	Cohort study	140	1188	March 1, 2020 to July 4, 2021	BNT162b2: 77.8% mRNA-1273: 12.9% Oxford-AstraZeneca: 9.3%	≥1 doses	Second trimester: 14.3% third trimester: 85.7%	Ultrasound and assisted reproductive technology	NA
Citu et al., 2022^24^	Romania	Cohort study	173	529	1 May 2021 to 1 August 2021	BNT162b2:66.4% Ad26.COV2.S:33.6%	BNT16b2:2 doses Ad26.COV2.S:1 dose	Third trimester	NA	NA
Dick (booster dose) et al.^a^, 2022^25^	Israel	Cohort study	3139	3368	July to October 2021	BNT162b2 or mRNA-1273	2 doses: 90.6%; 3 doses: 9.4%	During pregnancy	Ultrasound	No
Dick et al., 2022^26^	Israel	Cohort study	2305	3313	December 2020 to July 2021	BNT162b2	≥1 doses	First trimester: 0.5% second trimester: 41.8% third trimester: 57.7%	Menstrual period or ultrasound	No
Fell et al., 2022^27^	Canada	Cohort study	22660	74,930	December 14, 2020, to September 30, 2021	BNT162b2: 79.9% mRNA-1273: 19.9% Other:1%	1 dose: 51.7%; 2 doses: 48.3%	First trimester: 0.9% second trimester: 35.5% third trimester: 63.6%	Menstrual period	Yes
Goldshtein et al., 2021^28^	Israel	Cohort study	1387	1427	December 19, 2020, to February 28, 2021	BNT162b2	≥1 doses	During pregnancy	Menstrual period	NA
Goldshtein et al., 2022^29^	Israel	Cohort study	16738	7452	March 1, 2021, to September 31, 2021	BNT162b2	≥1 doses	First trimester: 11.1% second trimester: 53.7% third trimester: 35.2%	NA	No
Halasa et al., 2022^30^	US	Case control	234	815	July 1, 2021, to March 8, 2022	BNT162b2 or mRNA-1273	Two doses	NA	NA	NA
Lipkind et al., 2022^31^	US	Cohort study	10064	36,015	December 15, 2020 to July 22, 2021	BNT162b2: 54.4% mRNA-1273: 41.4% Ad26.COV2.S: 4.2%	mRNA: 1 dose: 51.7%; 2 doses: 48.3%	First trimester: 1.7%, second trimester: 36.4% third trimester: 61.8%	Menstrual period	No
Magnus et al., 2022^32^	Sweden Norway	Cohort study	28506	129,015	January 1 2021 to January 12 2022 (Sweden), or January 15, 2022 (Norway)	BNT162b2: 71.7% mRNA-1273:26.7% AZD1222:1.7%	1 dose: 24%; 2 doses: 76%	First trimester: 3.9% second trimester: 46.1% third trimester: 50.5%	Menstrual period or ultrasound	No
Peretz‐machluf et al., 2022^33^	Israel	Cohort study	3240	460	March to July 2021	BNT162b2	≥1 doses	During pregnancy	NA	No
Rottenstreich et al., 2022^34^	Israel	Cohort study	712	1063	January to April 2021	BNT162b2	2 doses	Third trimester	NA	Yes
Theiler et al., 2021^35^	US	Cohort study	140	1862	December 10 2020 to April 19, 2021	BNT162b2: 90.7% mRNA-1273: 8.6% Ad26.COV2.S: 0.7%	≥1 doses	During pregnancy	NA	NA
Wainstock et al., 2021^36^	Israel	Cohort study	913	3486	January to June 2021	BNT162b2	≥1 doses	Second or third trimester	NA	No

*NA* not applicable.

^a^Because refs. ^25^ and ^26^ have the same first author and published year, we have make a distinction by adding “booster dose” based on the characteristic of these studies.

### Meta-analysis of main neonatal outcomes

**Fig. 2:**
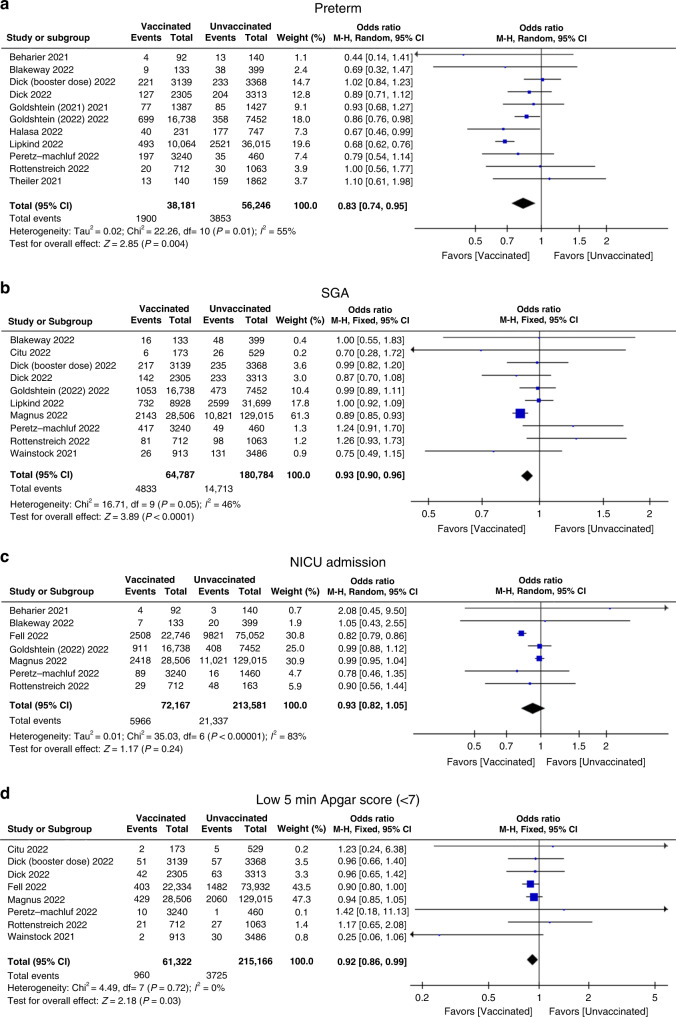
Odds ratio of main neonatal outcomes when comparing maternal COVID-19 vaccination versus unvaccination during pregnancy. Forest plots showing odds ratio of **a** preterm, **b** small for gestation (SGA), **c** NICU admission, and **d** low Apgar score at 5 min (<7). Vertical ticks within the blue boxes and horizontal lines show the mean effect and 95% confidence intervals for each study. Black diamond at the bottom shows the cumulative effect with 95% confidence intervals.

Of the 15 studies, 11 studies reported COVID-19 vaccination during pregnancy on preterm as a neonatal outcome, involving 38,181 vaccinated pregnant women and 56,246 unvaccinated pregnant women.^22,23,25,26,28–31,33–35^ The pooled OR (95% CI) was 0.83 (0.74, 0.95), with an overall effect *P* = 0.004. *I*^2^ = 55% (Fig. 2a and Table 2), which showed that the vaccine was beneficial by decreasing the rate of preterm birth. For SGA, 10 studies included this neonatal outcome.^23–26,29,31–34,36^ A 7% decrease in the odds of SGA was associated with COVID-19 vaccination when compared with unvaccination during pregnancy (pooled OR 0.93; 95% CI 0.90, 0.96, 64,787 vaccinated vs. 180,784 unvaccinated, *P* < 0.0001, *I*^2^ = 46%) (Fig. 2b and Table 2). The results also suggested that there is a protective effect of COVID-19 vaccination during pregnancy. Regarding NICU admission, a total of 7 studies involving 76,127 vaccinated pregnant women and 213,581 unvaccinated pregnant women were assessed, with pooled OR 0.93, 95% CI, 0.82, 1.05, *P* = 0.24, *I*^2^ = 83% (Fig. 2c and Table 2).^22,23,27,29,32–34^ There was no significant impact of COVID-19 vaccination during pregnancy on the odds of NICU admission. For the low 5 min Apgar score (<7), an 8% decrease in the odds of SGA was associated with COVID-19 vaccination when compared with unvaccination during pregnancy (8 studies pooled OR 0.92; 95% CI 0.86, 0.99, 61,322 vaccinated vs. 215,166 unvaccinated, *P* = 0.03, *I*^2^ = 0%) (Fig. 2d and Table 2).^24–27,32–34,36^ This result also indicated that COVID-19 vaccination could benefit by decreasing the odds of low Apgar score at 5 min.

**Table 2:** Meta-analysis of main neonatal outcomes in studies comparing COVID-19 vaccinated and unvaccinated pregnancies.

Neonatal outcomes	Number of studies	Vaccinated	Unvaccinated	Effect estimate	*P* value	*I*^2^%
Preterm birth	11	38,181	56,246	0.83 (0.74, 0.95)	0.004	55
SGA	10	64,787	180,784	0.93 (0.90, 0.96)	<0.0001	46
NICU admission	7	72,167	213,581	0.93 (0.82, 1.05)	0.24	83
Low 5 min Apgar score (<7)	8	61,322	215,166	0.92 (0.86, 0.99)	0.03	0

### Meta-analysis of additional neonatal outcomes

**Fig. 3:**
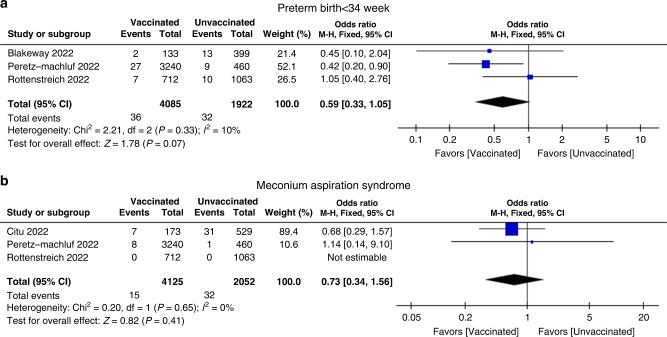
Odds ratio of additional neonatal outcomes when comparing maternal COVID-19 vaccination versus unvaccination during pregnancy. Forest plots showing odds ratio of **a** preterm birth with gestation <34 weeks and **b** meconium aspiration syndrome. Vertical ticks within the blue boxes and horizontal lines show the mean effect and 95% confidence intervals for each study. Black diamond at the bottom shows the cumulative effect with 95% confidence intervals.

To deeply examine the effect of COVID-19 vaccination in neonates, we also extracted other neonatal outcomes from all 15 studies, such as preterm birth with gestation <34 weeks, jaundice, meconium aspiration syndrome, hypoglycemia, sepsis, and transient tachypnea of the newborn. As shown in Table 3, these additional neonatal outcomes were assessed with pooled meta-analysis. In general, few reported data about these additional neonatal outcomes were provided from original studies. There were 2 additional neonatal outcomes, preterm birth with gestation <34 weeks and meconium aspiration syndrome, pooled analysis from 3 studies involving 4085, 4125 vaccinated pregnant women and 1922, 2052 unvaccinated pregnant women (OR = 0.59, 95% CI, 0.33–1.05 and OR = 0.73, 95% CI, 0.34–1.56), respectively (Fig. 3 and Table 3).^23,24,33,34^ In addition, other neonatal outcomes were pooled and analyzed from 2 studies, and all of the results are summarized in Table 3. The risks of encephalopathy, intracranial hemorrhage and respiratory distress syndrome were not assessable due to the low number of studies and insufficient data. According to the results from the pooled meta-analysis, there were no significant differences between COVID-19 vaccinated and unvaccinated pregnant women in any of the additional neonatal outcomes, which suggested that COVID-19 vaccination did not increase any adverse risk for these additional outcomes.

**Table 3:** Meta-analysis of additional neonatal outcomes in studies comparing COVID-19 vaccinated and unvaccinated pregnancies.

Neonatal outcomes	Number of studies	Vaccinated	Unvaccinated	Effect estimate	*P* value	*I*^2^%
Preterm birth <34 weeks	3	4085	1922	0.59 [0.33, 1.05]	0.07	10
Low birth weight <2500 g	2	16,878	9314	0.90 [0.79, 1.02]	0.10	0
Very low birth weight <1500 g	2	16,878	9314	0.79 [0.18, 3.54]	0.76	82
Congenital anomalies	2	2165	3969	0.71 [0.47, 1.06]	0.09	0
Jaundice	2	17,450	8515	0.84 [0.36, 1.95]	0.68	86
Meconium aspiration syndrome	3	4125	2052	0.73 [0.34, 1.56]	0.40	0
Mechanical ventilation	2	3952	1523	0.51 [0.26, 1.00]	0.05	0
Hypoglycemia	2	3952	1523	0.84 [0.58, 1.20]	0.33	0
Transient tachypnea of the newborn	2	3952	1523	0.66 [0.34, 1.27]	0.21	0
Fever	2	1086	4015	1.04 [0.34, 3.20]	0.94	0

### Heterogeneity

As shown in Table 2, preterm and NICU admission had moderate and high heterogeneity, with *I*^2^ = 55 and 83%, respectively. For Preterm, statistical heterogeneity was observed, which was attributable to Lipkind et al. study.^31^ After excluding data from this study, the pooled meta-analysis result showed that *I*^2^ = 0% (pooled OR 0.89; 95% CI 0.81, 0.96, 28,117 vaccinated vs. 20,231 unvaccinated, *P* = 0.005) (Supplemental Fig. 1). However, the overall effect did not change with *P* < 0.05, which still suggested that COVID-19 vaccination during pregnancy could decrease the odds of preterm birth. For NICU admission, statistical heterogeneity was observed, which was attributable to the Fell et al. study.^27^ After excluding data from this study, the pooled meta-analysis result showed that *I*^2^ = 0% (pooled OR 0.99; 95% CI 0.95, 1.03, 49,421 vaccinated vs. 138,529 unvaccinated, *P* = 0.68) (Supplemental Fig. 1). However, the overall effect did not change, with *P* = 0.68.

### Subgroup analysis

**Fig. 4:**
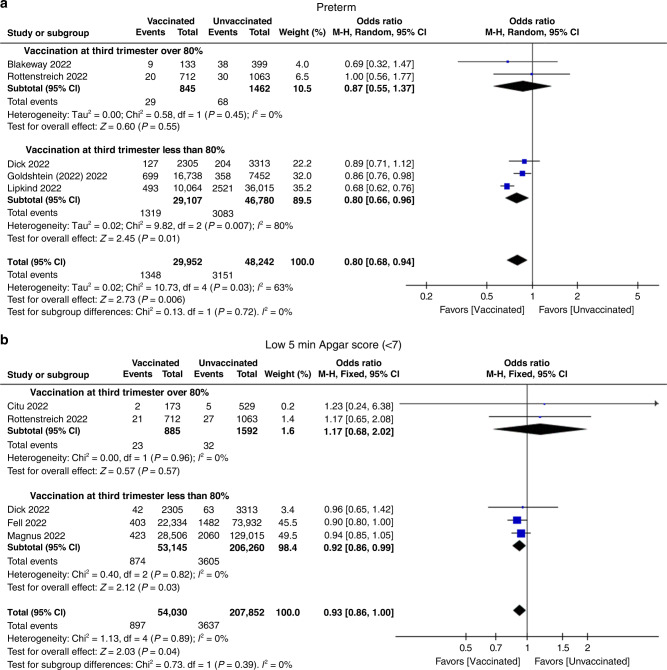
Subgroup analysis in term of percentage of vaccination during pregnancy at third trimester over 80% or not. Forest plots showing odds ratio of **a** preterm and **b** low Apgar score at 5 min (<7). Vertical ticks within the blue boxes and horizontal lines show the mean effect and 95% confidence intervals for each study. Black diamond at the bottom shows the cumulative effect with 95% confidence intervals.

We conducted a subgroup analysis of the main neonatal outcomes based on the timing of vaccination. In all of these included studies, few COVID-19 vaccinations occurred in the first trimester. Most vaccinations occurred in the second and third trimesters. We have subgrouped these data as a percentage of vaccination in the third trimester over 80% or not. As shown in Fig. 4a, vaccination in trimesters >80% had no effect on preterm infants (pooled OR 0.87; 95% CI 0.55, 1.37, *P* = 0.55, *I*^2^ = 0%), while vaccination in trimesters <80% had a protective effect on preterm infants (pooled OR 0.80; 95% CI 0.66, 0.96, *P* = 0.01, *I*^2^ = 80%). Additionally, vaccination in trimesters >80% had no effect on the low 5 min Apgar score (pooled OR 1.17; 95% CI 0.68, 2.02, *P* = 0.57, *I*^2^ = 0%), while vaccination in trimesters <80% had a protective effect on the Low 5 min Apgar score (pooled OR 0.92; 95% CI 0.86, 0.99, *P* = 0.03, *I*^2^ = 0%) (Fig. 4b).

## Discussion

This systematic review and meta-analysis summarized current data on neonatal outcomes after maternal COVID-19 vaccination based on observational studies. This meta-analysis consisted of 15 studies. We found that newborns would benefit from maternal vaccination of COVID-19 with a lower incidence of preterm, SGA, and low 5 min Apgar score (<7). In addition, there was no increased risk of NICU admission or other additional neonatal outcomes, such as low birth weight, meconium aspiration syndrome, mechanical ventilation, hypoglycemia, and so on. Importantly, limited data seem to identify that vaccination with COVID-19 before the third trimester may have a protective effect on preterm as well as low 5 min Apgar score.

Due to concerns about the adverse maternal and neonatal outcomes, there is still a high rate of hesitancy among pregnant women to accept COVID-19 vaccination.^38,39^ Initially, there were few clinical data about the safety and effectiveness of COVID-19 vaccination during pregnancy. Then, as time passes, due to high COVID-19-related morbidity and mortality among pregnant women as well as their neonates, several countries have recommended COVID-19 vaccination during pregnancy.^16–18^ Although there are few RCT clinical studies, real-world studies about the safety and effectiveness of COVID-19 vaccines for pregnant women have already suggested that vaccination has protective effects on the prevention of COVID-19 infection without increasing the risk of adverse perinatal outcomes.^12–15^ Among these data, most studies focused on maternal outcomes, while neonatal outcomes were reported in a small portion. Recently, several studies about neonatal and early infant outcomes after maternal COVID-19 vaccination have already received increasing attention.^12–15,22–36^ However, a comprehensive meta-analysis of neonatal outcomes after maternal COVID-19 vaccination is still lacking.

Thus, we conducted a systematic review and meta-analysis of published data mainly concerning neonatal outcomes after maternal COVID-19 vaccination. In the present study, the main neonatal outcomes are preterm, SGA, NICU admission, and low 5 min Apgar score (<7). In population-based larger cohorts published from Israel, Canada, Sweden, and Norway, and the US, except for data from Canada, all three other studies reported that COVID-19 vaccination during pregnancy was not significantly associated with an increased risk for adverse neonatal outcomes.^27,29,31,32^ After pooling all of these data, our results showed not only no increased risk from maternal vaccination but also lower rates of adverse neonatal outcomes among those born to individuals vaccinated during pregnancy. As shown in Fig. 2 and Table 2, 17%, 7%, and 8% decreases in the odds of preterm, SGA, and low 5 min Apgar score (<7) were associated with COVID-19 vaccination when compared with unvaccination during pregnancy, respectively. These results may gain confidence to improve the acceptance of vaccines among pregnant women.

However, except for the main neonatal outcomes, more detailed additional neonatal outcomes could only be extracted from 4 studies.^23,24,33,34^ Although limited data were acquired from these studies, most of these variables presented lower heterogeneity after pooling together. As shown in Table 3, there was no increased risk from maternal vaccination in any of these neonatal outcomes.

In all of 15 included studies, there are two COVID-19 vaccine platforms, the mRNA vaccine and the viral vector vaccine involved in included studies. Most of individuals were vaccinated with mRNA COVID-19 in included studies, though mRNA COVID-19 vaccine have already been proven to have high efficacy and to protect against different variants or strains, the major concern still persists about the uncertainty of long term adverse effect due to these are the first approved mRNA vaccines to date. As for viral vector vaccines, they have high gene transduction efficiency and high specificity of genes delivered to target cells while the current data showed the low efficacy against COVID-19.^40^

In most of the included studies, pregnant women received COVID-19 vaccination in the second and third trimesters, and few data were acquired from neonates’ vaccination exposure in the first trimester. Due to antibody and cellular immune responses occurring at the early stages after vaccination, there is a question about whether vaccination at early stages of pregnancy could increase the risk of miscarriage, fetal anomalies or adverse neonatal outcomes. In previous population-based studies, no increased miscarriage risk was found after COVID-19 vaccination during early pregnancy.^41,42^ However, very few studies reported neonatal outcomes according to trimester at vaccination. Thus, in the present study, based on the timing of exposure vaccination, we have subgroup studies as the vast majority of exposure vaccinations in the third trimester and the remaining part. The subgroup meta-analysis showed that vaccination before the third trimester could benefit neonates with lower rates of preterm birth, while there was neither an adverse effect nor a beneficial effect on preterm infants in the group that were primarily vaccinated in the third trimester. In fact, pregnancy received at the third trimester means that this cohort of pregnant women had already been at a late stage of pregnancy and had already excluded some preterm births in this cohort. Thus, there may be a risk of bias in this cohort. We still need future clinical studies to examine neonatal outcomes of COVID-19 vaccination based on each stage of pregnancy.

The present study has several limitations. First, due to limited data, the absence of randomized controlled trials was the biggest limitation of our meta-analysis. Second, neonatal outcomes based on different doses of vaccine, different types of vaccine and detailed trimesters at vaccination could not be deeply examined. Third, the additional neonatal outcomes were challenged by low numbers of studies. Fourth, all studies included data from developed countries, and most vaccines were mRNA vaccines, but data were not acquired from developing countries and other types of vaccines, which does not represent the true condition worldwide. Fifth, different strains could have different effect on neonatal outcomes,^43,44^ due to limited information from primary data, we could not identify if there are different neonatal outcomes after maternal vaccination of COVID-19 during different strains pandemic. Even so, these limitations could encourage researchers to examine the effect of COVID-19 vaccination on neonatal outcomes according to these aspects.

The present data not only showed that COVID-19 vaccination in pregnant women does not raise significant adverse effects on neonatal outcomes but also confirmed the benefits of vaccination during pregnancy on neonatal outcomes. These neonatal outcomes including in present study focus on the adverse side. Actually, trans-placental transfer of maternal neutralizing antibodies to newborn and existence antibodies in breast milk have already been reported by several studies.^13,20^ These data suggest that vaccination of COVID-19 in pregnant would not only make immunity to the pregnant individual but may also indicated a potential protective effect on the infection and vaccination of newborn.

Infection of COVID-19 has been proven to produce several adverse outcomes on pregnant women. Vaccination of COVID-19 in pregnant women is still effective method for prevention infection. However, how to relieve pregnant women’s concerns on vaccination is one of important problem during vaccination. Though pregnant women were excluded from participation in initial COVID-19 vaccine trials, the persisted pandemic of COVID-19 and COVID-19 related morbidity and mortality during this population contribute to vaccination of COVID-19 in some pregnant individuals.^29^ And also some national or regional policy or strategies recommends pregnant receive a COVID- 19 vaccine.^28,29^ More and more data have showed benefits of COVID-19 vaccination outweigh the risks during pregnancy.^22–36^ In return, professional recommendation based on these data could contribute to relieve pregnant women’s concerns on vaccination. By now, the American College of Obstetricians and Gynecologists, European Board and College of Obstetrics and Gynecology, Israel Ministry of Health and so on have already recommended pregnant receive a COVID-19 vaccine.^5,17,18^ In present study, we have included 15 studies. As results, the present data not only showed that COVID-19 vaccination in pregnant women does not raise significant adverse effects on neonatal outcomes but also confirmed the benefits of vaccination during pregnancy on neonatal outcomes. Our studies could provide additional data to support and improve these strategies and improve the acceptance of vaccines among pregnant women.

## Supplementary information

supplemental figure 1

Supplemental methods

Supplemental table 1 PRISMA_2020_checklist

Supplemental table 2 ROBINS-I

## Data Availability

The data described in this manuscript are contained in published articles or available from the corresponding author upon reasonable request.
