# Supplementary material for: Systematic review and meta-analysis of neonatal outcomes of COVID-19 vaccination in pregnancy
Source: Pediatr Res. 2023 Jan 3;94(1):34–42. doi: 10.1038/s41390-022-02421-0 (PMC9808682; doi:10.1038/s41390-022-02421-0)
Supplement: Supplementary file 1 — supplemental figure 1 [file 41390_2022_2421_MOESM1_ESM.pdf]

Supplemental Figure

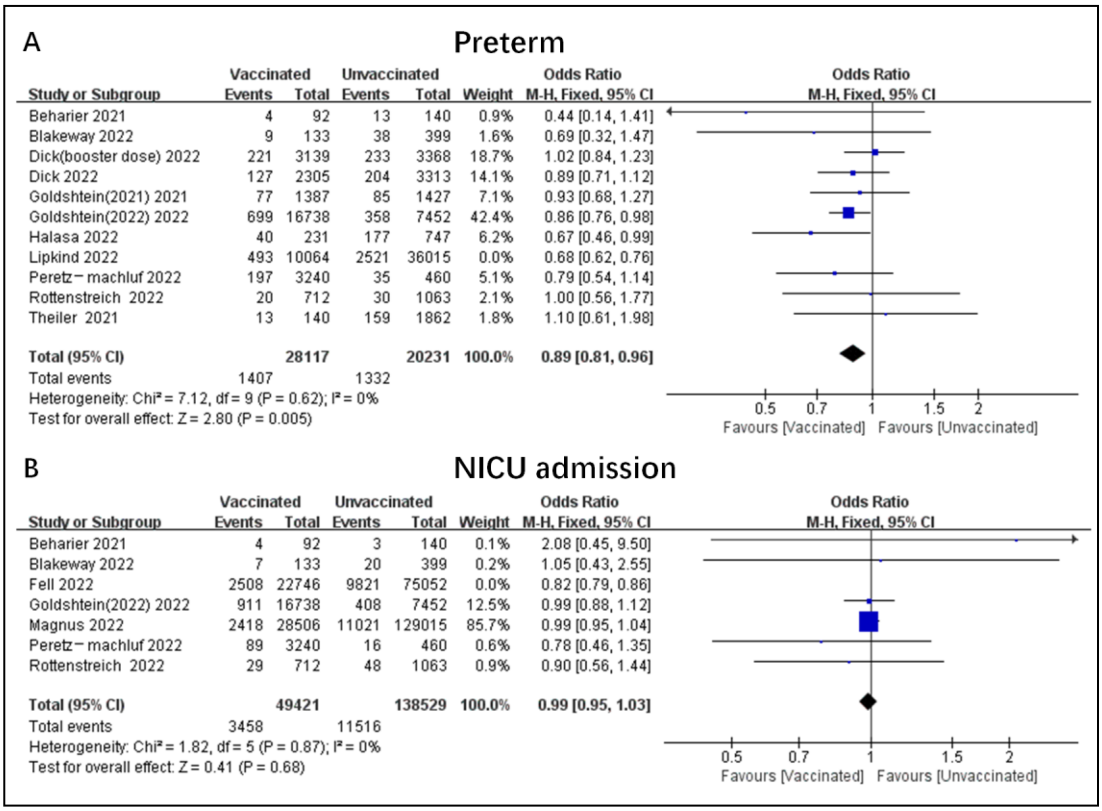

Supplemental Figure 1. Heterogeneity analysis of the main neonatal outcomes was performed by excluding each included study (heterogeneity analysis was performed when $I^2 \geq 50\%$). (A) Odds ratio of Preterm when comparing maternal COVID-19 vaccination versus unvaccination during pregnancy after excluding Lipkind et al study. (B) Odds ratio of SGA when comparing maternal COVID-19 vaccination versus unvaccination during pregnancy after excluding the Fell et al study.
