# Supplementary material for: Systematic review and meta-analysis of neonatal outcomes of COVID-19 vaccination in pregnancy
Source: Pediatr Res. 2023 Jan 3;94(1):34–42. doi: 10.1038/s41390-022-02421-0 (PMC9808682; doi:10.1038/s41390-022-02421-0)
Supplement: Supplementary file 2 — Supplemental methods [file 41390_2022_2421_MOESM2_ESM.pdf]

Search strategy

Pubmed

1. ("Pregnancy"[Mesh])
2. (Pregnancies[Title/Abstract]) OR (Gestation[Title/Abstract])
3. 1 or 2
4. (“Infant, Newborn” [Mesh])
5. (Infants, Newborn[Title/Abstract]) OR (Newborn Infant[Title/Abstract]) OR (Newborn Infants[Title/Abstract]) OR (Newborns[Title/Abstract]) OR (Newborn[Title/Abstract]) OR (Neonate[Title/Abstract]) OR (Neonates[Title/Abstract])
6. 4 or 5
7. (“COVID-19 Vaccines”[Mesh])
8. (COVID 19 Vaccines[Title/Abstract]) OR (Vaccines, COVID-19[Title/Abstract]) OR (COVID-19 Virus Vaccines[Title/Abstract]) OR (COVID 19 Virus Vaccines[Title/Abstract]) OR (Vaccines, COVID-19 Virus[Title/Abstract]) OR (Virus Vaccines, COVID-19[Title/Abstract]) OR (COVID-19 Virus Vaccine[Title/Abstract]) OR (COVID 19 Virus Vaccine[Title/Abstract]) OR (Vaccine, COVID-19 Virus[Title/Abstract]) OR (Virus Vaccine, COVID-19[Title/Abstract]) OR (COVID19 Virus Vaccines[Title/Abstract]) OR (Vaccines, COVID19 Virus[Title/Abstract]) OR (Virus Vaccines, COVID19[Title/Abstract]) OR (COVID19 Virus Vaccine[Title/Abstract]) OR (Vaccine, COVID19 Virus[Title/Abstract]) OR (Virus Vaccine, COVID19[Title/Abstract]) OR (COVID19 Vaccines[Title/Abstract]) OR (Vaccines, COVID19[Title/Abstract]) OR (COVID19 Vaccine[Title/Abstract]) OR (Vaccine, COVID19[Title/Abstract]) OR (SARS-CoV-2 Vaccines[Title/Abstract]) OR (SARS CoV 2 Vaccines[Title/Abstract]) OR (Vaccines, SARS-CoV-2[Title/Abstract]) OR (SARS-CoV-2 Vaccine[Title/Abstract]) OR (SARS CoV 2 Vaccine[Title/Abstract]) OR (Vaccine, SARS-CoV-2[Title/Abstract]) OR (SARS2 Vaccines[Title/Abstract]) OR (Vaccines, SARS2[Title/Abstract])) OR (SARS2 Vaccine[Title/Abstract])) OR (Vaccine, SARS2[Title/Abstract]) OR (Coronavirus Disease 2019 Vaccines[Title/Abstract]) OR (Coronavirus Disease 2019 Vaccine[Title/Abstract]) OR (Coronavirus Disease 2019 Virus Vaccine[Title/Abstract]) OR (Coronavirus Disease 2019 Virus Vaccines[Title/Abstract]) OR (Coronavirus Disease-19 Vaccines[Title/Abstract]) OR (Coronavirus Disease 19 Vaccines[Title/Abstract]) OR (Vaccines, Coronavirus Disease-19[Title/Abstract]) OR (Coronavirus Disease-19 Vaccine[Title/Abstract]) OR (Coronavirus Disease 19 Vaccine[Title/Abstract]) OR (Vaccine, Coronavirus Disease-19[Title/Abstract]) OR (COVID 19 Vaccine[Title/Abstract]) OR (Vaccine, COVID 19[Title/Abstract]) OR (2019-nCoV Vaccine[Title/Abstract]) OR (2019 nCoV Vaccine[Title/Abstract]) OR (Vaccine, 2019-

nCoV[Title/Abstract]) OR (2019 Novel Coronavirus Vaccines[Title/Abstract]) OR (2019 Novel Coronavirus Vaccine[Title/Abstract]) OR (2019-nCoV Vaccines[Title/Abstract]) OR (2019 nCoV Vaccines[Title/Abstract]) OR (Vaccines, 2019-nCoV[Title/Abstract]) OR (COVID-19 Vaccine[Title/Abstract]) OR (Vaccine, COVID-19[Title/Abstract]) OR (SARS Coronavirus 2 Vaccines[Title/Abstract])
9. 7 or 8
10. (“COVID-19”[MeSH])
11. (COVID 19[Title/Abstract]) OR (SARS-CoV-2 Infection[Title/Abstract]) OR (Infection, SARS-CoV-2[Title/Abstract]) OR (SARS CoV 2 Infection[Title/Abstract]) OR (SARS-CoV-2 Infections[Title/Abstract]) OR (2019 Novel Coronavirus Disease[Title/Abstract]) OR (2019 Novel Coronavirus Infection[Title/Abstract]) OR (2019-nCoV Disease[Title/Abstract]) OR (2019 nCoV Disease[Title/Abstract]) OR (2019-nCoV Diseases[Title/Abstract]) OR (Disease, 2019-nCoV[Title/Abstract]) OR (COVID-19 Virus Infection[Title/Abstract]) OR (COVID 19 Virus Infection[Title/Abstract]) OR (COVID-19 Virus Infections[Title/Abstract]) OR (Infection, COVID-19 Virus[Title/Abstract]) OR (Virus Infection, COVID-19[Title/Abstract]) OR (Coronavirus Disease 2019[Title/Abstract]) OR (Disease 2019, Coronavirus[Title/Abstract]) OR (Coronavirus Disease-19[Title/Abstract]) OR (Coronavirus Disease 19[Title/Abstract]) OR (Severe Acute Respiratory Syndrome Coronavirus 2 Infection[Title/Abstract]) OR (SARS Coronavirus 2 Infection[Title/Abstract]) OR (COVID-19 Virus Disease[Title/Abstract]) OR (COVID 19 Virus Disease[Title/Abstract]) OR (COVID-19 Virus Diseases[Title/Abstract]) OR (Disease, COVID-19 Virus[Title/Abstract]) OR (Virus Disease, COVID-19[Title/Abstract]) OR (2019-nCoV Infection[Title/Abstract]) OR (2019 nCoV Infection[Title/Abstract]) OR (2019-nCoV Infections[Title/Abstract]) OR (Infection, 2019-nCoV[Title/Abstract]) OR (COVID19[Title/Abstract]) OR (COVID-19 Pandemic[Title/Abstract]) OR (COVID 19 Pandemic[Title/Abstract]) OR (Pandemic, COVID-19[Title/Abstract]) OR (COVID-19 Pandemics[Title/Abstract])
12. 10 or 11
13. (“Vaccines”[MeSH])
14. Vaccine[Title/Abstract]
15. 13 or 14
16. 12 and 15
17. 9 or 16
18. 3 and 6 and 17

Embase

1. 'pregnancy'/exp
2. pregnancies:ab,ti OR gestation:ab,ti
3. 1 or 2
4. 'infant, newborn'/exp
5. 'infants, newborn':ab,ti OR 'newborn infant':ab,ti OR 'newborn infants':ab,ti OR newborns:ab,ti OR newborn:ab,ti OR neonate:ab,ti OR neonates:ab,ti
6. 4 or 5
7. 'covid-19 vaccines'/exp
8. 'covid 19 vaccines':ab,ti OR 'vaccines, covid-19':ab,ti OR 'covid-19 virus vaccines':ab,ti OR 'covid 19 virus vaccines':ab,ti OR 'vaccines, covid-19 virus':ab,ti OR 'virus vaccines, covid-19':ab,ti OR 'covid-19 virus vaccine':ab,ti OR 'covid 19 virus vaccine':ab,ti OR 'vaccine, covid-19 virus':ab,ti OR 'virus vaccine, covid-19':ab,ti OR 'covid19 virus vaccines':ab,ti OR 'vaccines, covid19 virus':ab,ti OR 'virus vaccines, covid19':ab,ti OR 'covid19 virus vaccine':ab,ti OR 'vaccine, covid19 virus':ab,ti OR 'virus vaccine, covid19':ab,ti OR 'covid19 vaccines':ab,ti OR 'vaccines, covid19':ab,ti OR 'covid19 vaccine':ab,ti OR 'vaccine, covid19':ab,ti OR 'sars-cov-2 vaccines':ab,ti OR 'sars cov 2 vaccines':ab,ti OR 'vaccines, sars-cov-2':ab,ti OR 'sars-cov-2 vaccine':ab,ti OR 'sars cov 2 vaccine':ab,ti OR 'vaccine, sars-cov-2':ab,ti OR 'sars2 vaccines':ab,ti OR 'vaccines, sars2':ab,ti OR 'sars2 vaccine':ab,ti OR 'vaccine, sars2':ab,ti OR 'coronavirus disease 2019 vaccines':ab,ti OR 'coronavirus disease 2019 vaccine':ab,ti OR 'coronavirus disease 2019 virus vaccine':ab,ti OR 'coronavirus disease 2019 virus vaccines':ab,ti OR 'coronavirus disease-19 vaccines':ab,ti OR 'coronavirus disease 19 vaccines':ab,ti OR 'vaccines, coronavirus disease-19':ab,ti OR 'coronavirus disease-19 vaccine':ab,ti OR 'coronavirus disease 19 vaccine':ab,ti OR 'vaccine, coronavirus disease-19':ab,ti OR 'covid 19 vaccine':ab,ti OR 'vaccine, covid 19':ab,ti OR '2019-ncov vaccine':ab,ti OR '2019 ncov vaccine':ab,ti OR 'vaccine, 2019-ncov':ab,ti OR '2019 novel coronavirus vaccines':ab,ti OR '2019 novel coronavirus vaccine':ab,ti OR '2019-ncov vaccines':ab,ti OR '2019 ncov vaccines':ab,ti OR 'vaccines, 2019-ncov':ab,ti OR 'covid-19 vaccine':ab,ti OR 'vaccine, covid-19':ab,ti OR 'sars coronavirus 2 vaccines':ab,ti
9. 7 or 8
10. 'covid-19'/exp
11. 'covid 19':ab,ti OR 'sars-cov-2 infection':ab,ti OR 'infection, sars-cov-2':ab,ti OR 'sars cov 2 infection':ab,ti OR 'sars-cov-2 infections':ab,ti OR '2019 novel coronavirus disease':ab,ti OR '2019 novel coronavirus infection':ab,ti OR '2019-ncov disease':ab,ti OR '2019 ncov disease':ab,ti OR '2019-ncov diseases':ab,ti OR 'disease, 2019-ncov':ab,ti OR 'covid-19 virus infection':ab,ti OR 'covid 19 virus infection':ab,ti OR 'covid-19 virus infections':ab,ti OR 'infection, covid-19 virus':ab,ti OR 'virus infection, covid-19':ab,ti OR 'coronavirus disease

2019':ab,ti OR 'disease 2019, coronavirus':ab,ti OR 'coronavirus disease-19':ab,ti OR 'coronavirus disease 19':ab,ti OR 'severe acute respiratory syndrome coronavirus 2 infection':ab,ti OR 'sars coronavirus 2 infection':ab,ti OR 'covid-19 virus disease':ab,ti OR 'covid 19 virus disease':ab,ti OR 'covid-19 virus diseases':ab,ti OR 'disease, covid-19 virus':ab,ti OR 'virus disease, covid-19':ab,ti OR '2019-ncov infection':ab,ti OR '2019 ncov infection':ab,ti OR '2019-ncov infections':ab,ti OR 'infection, 2019-ncov':ab,ti OR covid19:ab,ti OR 'covid-19 pandemic':ab,ti OR 'covid 19 pandemic':ab,ti OR 'pandemic, covid-19':ab,ti OR 'covid-19 pandemics':ab,ti
12. 10 or 11
13. 'vaccines'/exp
14. vaccine:ab,ti
15. 13 or 14
16. 12 and 15
17. 9 or 16
18. 3 and 6 and 17

WHO Covid-19 database

1. ((pregnancy) OR (pregnancies) OR (gestation)) [Title/Abstract/Subject]
2. ((infant, newborn) OR (infants, newborn) OR (newborn infant) OR (newborn infants) OR (newborns) OR (newborn) OR (neonate) OR (neonates)) [Title/Abstract/Subject]
3. ((covid-19 vaccines) OR (covid 19 vaccines) OR (vaccines, covid-19) OR (covid-19 virus vaccines) OR (covid 19 virus vaccines) OR (vaccines, covid-19 virus) OR (virus vaccines, covid-19) OR (covid-19 virus vaccine) OR (covid 19 virus vaccine) OR (vaccine, covid-19 virus) OR (virus vaccine, covid-19) OR (covid19 virus vaccines) OR (vaccines, covid19 virus) OR (virus vaccines, covid19) OR (covid19 virus vaccine) OR (vaccine, covid19 virus) OR (virus vaccine, covid19) OR (covid19 vaccines) OR (vaccines, covid19) OR (covid19 vaccine) OR (vaccine, covid19) OR (sars-cov-2 vaccines) OR (sars cov 2 vaccines) OR (vaccines, sars-cov-2) OR (sars-cov-2 vaccine) OR (sars cov 2 vaccine) OR (vaccine, sars-cov-2) OR (sars2 vaccines) OR (vaccines, sars2) OR (sars2 vaccine) OR (vaccine, sars2) OR (coronavirus disease 2019 vaccines) OR (coronavirus disease 2019 vaccine) OR (coronavirus disease 2019 virus vaccine) OR (coronavirus disease 2019 virus vaccines) OR (coronavirus disease-19 vaccines) OR (coronavirus disease 19 vaccines) OR (vaccines, coronavirus disease-19) OR (coronavirus disease-19 vaccine) OR (coronavirus disease 19 vaccine) OR (vaccine, coronavirus disease-19) OR (covid 19 vaccine) OR (vaccine, covid 19) OR (2019-ncov vaccine) OR (2019 ncov vaccine) OR (vaccine, 2019-ncov) OR (2019 novel coronavirus vaccines) OR (2019 novel coronavirus vaccine) OR (2019-ncov vaccines) OR (2019 ncov vaccines) OR (vaccines, 2019-ncov) OR (covid-19 vaccine) OR (vaccine, covid-19) OR (sars coronavirus 2 vaccines)) [Title/Abstract]
4. ((covid-19) OR (covid 19) OR (sars-cov-2 infection) OR (infection, sars-cov-2) OR (sars cov 2 infection) OR (sars-cov-2 infections) OR (2019 novel coronavirus disease) OR (2019 novel coronavirus infection) OR (2019-ncov disease) OR (2019 ncov disease) OR (2019-ncov diseases) OR (disease, 2019-ncov) OR (covid-19 virus infection) OR (covid 19 virus infection) OR (covid-19 virus infections) OR (infection, covid-19 virus) OR (virus infection, covid-19) OR (coronavirus disease 2019) OR (disease 2019, coronavirus) OR (coronavirus disease-19) OR (coronavirus disease 19) OR (severe acute respiratory syndrome coronavirus 2 infection) OR (sars coronavirus 2 infection) OR (covid-19 virus disease) OR (covid 19 virus disease) OR (covid-19 virus diseases) OR (disease, covid-19 virus) OR (virus disease, covid-19) OR (2019-ncov infection) OR (2019 ncov infection) OR (2019-ncov infections) OR (infection, 2019-ncov) OR covid19:ab,ti OR (covid-19 pandemic) OR (covid 19 pandemic) OR (pandemic, covid-19) OR (covid-19 pandemics)) [Title/Abstract/Subject]
5. ((vaccines) OR (vaccine)) [Title/Abstract/Subject]
6. 4 and 5

7. 3 or 6
8. 1 and 2 and 7
