# Supplementary material for: Systematic review and meta-analysis of neonatal outcomes of COVID-19 vaccination in pregnancy
Source: Pediatr Res. 2023 Jan 3;94(1):34–42. doi: 10.1038/s41390-022-02421-0 (PMC9808682; doi:10.1038/s41390-022-02421-0)
Supplement: Supplementary file 4 — Supplemental table 2 ROBINS-I [file 41390_2022_2421_MOESM4_ESM.pdf]

ROBINS-I risk of bias assessment of observational studies.

Author, year	Confounding	Selection bias	Bias in measurement classification of interventions	Bias due to deviations from intended interventions	Bias due to missing data	Bias in measurement of outcomes	Bias in selection of the reported result	Overall bias
Beharier et al, 2021	Moderate	Moderate	Low	Low	Low	Low	Low	Moderate
Blakeway et al, 2022	Moderate	Low	Low	Low	Low	Low	Low	Moderate
Citu et al, 2022	Moderate	Low	Low	Low	Low	Low	Low	Moderate
Dick (booster dose) et al, 2022	Moderate	Low	Low	Low	Low	Low	Low	Moderate
Dick et al, 2022	Moderate	Low	Low	Low	Low	Low	Low	Moderate
Fell et al, 2022	Moderate	Low	Low	Low	Low	Low	Low	Moderate
Goldshtein et al, 2021	Moderate	Low	Low	Low	Low	Low	Low	Moderate
Goldshtein et al, 2022	Moderate	Low	Low	Low	Low	Low	Low	Moderate
Halasa et al	Moderate	Low	Low	Low	Moderate	Low	Low	Moderate
Lipkind et al, 2022	Moderate	Low	Low	Low	Low	Low	Low	Moderate
Magnus et al, 2022	Moderate	Low	Low	Low	Low	Low	Low	Moderate

Peretz-machluf et al, 2022	Moderate	Low	Low	Low	Low	Low	Low	Moderate
Rottenstreich et al, 2022	Moderate	Low	Low	Low	Low	Low	Low	Moderate
Theiler et al, 2021	Moderate	Low	Low	Low	Low	Low	Low	Moderate
Wainstock et al, 2021	Moderate	Moderate	Low	Low	Low	Low	Low	Moderate

Low risk: The study is comparable to a well performed randomized trial; moderate risk: The study provides sound evidence for a non-randomized study but cannot be considered comparable to a well performed randomized trial; serious risk: The study has some important problem; critical risk: The study is too problematic to provide any useful evidence and should not be included in any synthesis.
